# Fluid resuscitation in adults with severe infection and sepsis: a systematic review and network meta-analysis

**DOI:** 10.3389/fmed.2025.1543586

**Published:** 2025-06-17

**Authors:** Binglin Song, Kangrui Fu, Xiangde Zheng, Chun Liu

**Affiliations:** ^1^Clinical Medical College of North Sichuan Medical College, Nanchong, China; ^2^Emergency Department of Dazhou Central Hospital, Dazhou, China

**Keywords:** fluid resuscitation, sepsis, colloids, crystalloids, meta-analysis

## Abstract

**Introduction:**

The choice of optimal resuscitation fluid for patients with septic shock remains a controversial topic. The 2021 Sepsis Surviving Campaign Guidelines strongly recommend using crystalloids as the first-line resuscitation fluid for adults with sepsis or septic shock, with balanced crystalloids as a weak recommendation. However, two large-scale network meta-analyses in 2020 concluded that balanced crystalloids are most advantageous. This study reevaluates the efficacy and safety of different resuscitation fluids in septic shock through a network meta-analysis (NMA).

**Methods:**

Databases including PubMed, EMBASE, and WOS were searched, and reference lists of relevant literature up to September 2024 were reviewed. Studies involving adult patients with sepsis requiring fluid resuscitation were selected. The fluids covered include balanced crystalloid (BC), saline, iso-oncotic albumin (Iso-Alb), hyper-oncotic albumin (Hyper-Alb), low molecular weight hydroxyethyl starch (L-HES), high molecular weight hydroxyethyl starch (HES), and gelatin. A network meta-analysis was conducted to assess the effects of different fluid types.

**Results:**

A total of 32 RCTs were included in the analysis. The NMA probability ranking results show that balanced crystalloid (BC) had the lowest all-cause mortality rate, with the highest SUCRA value (83.1%). Gelatin was shown to confer the greatest advantage in terms of kidney injury, with the highest SUCRA value (80.7%). Hyper-oncotic albumin had the lowest occurrence of renal replacement therapy events, showing the highest SUCRA value (94.1%). Patients treated with balanced crystalloids had the shortest ICU stays and hospital lengths of stay.

**Conclusion:**

Balanced solutions (BS) are the preferred resuscitation fluids for septic shock. High molecular weight hydroxyethyl starch (H-HES) is associated with increased risks of mortality, acute kidney injury (AKI), and renal replacement therapy (RRT), as well as prolonged hospital stays, and its use is advised against. Gelatin is associated with poorer outcomes in terms of mortality, continuous renal replacement therapy (CRRT), and length of hospital stay.

**Systematic review registration:**

Registration ID: INPLASY2024100049 https://doi.org/10.37766/inplasy2024.10.0049.

## Introduction

Sepsis is a systemic inflammatory response syndrome triggered by infection, often resulting in severe hypotension, metabolic disturbances, and ultimately, organ failure (1). If not promptly addressed, this pathological state can be life-threatening for patients. One of the significant challenges in treating sepsis is the effective management of fluid resuscitation, which is a core component of septic shock therapy. Fluid resuscitation works by increasing vascular volume, enhancing cardiac output, and improving tissue perfusion, all of which are critical for effective treatment (2). Generally, resuscitation fluids are classified into crystalloids and colloids. Common crystalloids, such as normal saline and lactated Ringer’s solution, rapidly replenish blood volume and augment cardiac output. Colloids (e.g., albumin and hydroxyethyl starch) use larger molecules to retain fluid in the bloodstream, making them suitable for maintaining hemodynamic stability over longer periods (3, 4).

Early resuscitation guidelines favored crystalloids, particularly normal saline (0.9% sodium chloride), as the standard fluid for treating critically ill patients. However, subsequent research has shown that the use of normal saline may lead to electrolyte imbalances and metabolic acidosis, negatively affecting patient outcomes (5). This led to a growing preference for balanced crystalloids, whose sodium and chloride concentrations are closer to physiological levels, thereby reducing the risk of acid–base imbalances (6). A 2014 network meta-analysis of 14 randomized controlled trials (RCTs) involving sepsis patients found that the use of balanced crystalloids, compared to normal saline, was associated with reduced mortality (4). The 2016 SALT trial (*n* = 974), which compared balanced solutions with normal saline, primarily assessed mortality, acute renal replacement therapy (RRT), and composite outcomes of sustained renal dysfunction, revealing no significant differences between the two groups. A 2018 single-center study involving 15,802 patients found that balanced solutions significantly reduced 30-day mortality compared to normal saline (OR 0.80; 95% CI 0.67–0.94) (7). Current guidelines recommend crystalloids as the first-line resuscitation fluid. The use of colloids has historically been controversial. Early studies suggested that colloids were more effective than crystalloids due to their larger molecular size, which allows them to remain in the vascular compartment longer, thereby maintaining blood volume. However, later research has shown that certain colloids, such as hydroxyethyl starch, may be associated with acute kidney injury (AKI) and other adverse reactions (8). Additionally, two single-center trials and two meta-analyses (9–12) found no significant difference in 30-day or 90-day mortality between albumin and crystalloids. The 2021 guidelines concluded that albumin is not the preferred fluid for resuscitation in patients with sepsis or septic shock and recommended against the use of gelatin and starch for sepsis resuscitation.

Following the publication of the 2021 guidelines, several studies on sepsis fluid resuscitation were conducted. One RCT involving 10,520 patients found that balanced solutions did not significantly reduce 90-day mortality compared to normal saline (13). A 2022 RCT with 5,037 patients found no significant differences in the incidence of adverse and severe adverse events between balanced solutions and normal saline (14). In 2024, an RCT involving 301 participants from 15 medical institutions demonstrated that balanced crystalloids resulted in lower mortality compared to 5% albumin (15). Recent large-scale RCTs have provided new evidence regarding the effectiveness of different resuscitation fluids, further advancing the search for the optimal resuscitation fluid (10).

The primary objective of this study is to comprehensively evaluate the efficacy and safety of different resuscitation fluids for patients with septic shock through a network meta-analysis (NMA). We aim to compare the effects of balanced crystalloids, normal saline, albumin, hydroxyethyl starch, and gelatin, and rank these resuscitation fluids based on the Surface Under the Cumulative Ranking Curve (SUCRA), thereby providing more valuable information for clinical decision-making. By reanalyzing the most recent randomized controlled trial (RCT) data, this study seeks to provide scientific evidence for selecting the optimal resuscitation fluid for patients with septic shock.

Traditional meta-analyses typically compare only two types of resuscitation fluids, limiting their ability to assess the effects of multiple fluid types simultaneously. Network meta-analysis (NMA) is a more advanced statistical method that allows comprehensive comparisons of all resuscitation fluids within the same analytical framework. NMA not only compares the relative effects of various resuscitation fluids but also ranks their effectiveness using the Surface Under the Cumulative Ranking Curve (SUCRA), providing more valuable information for clinical decision-making. By combining systematic reviews with NMA, it is possible to integrate both direct and indirect evidence to summarize the efficacy and safety of different resuscitation fluids in septic shock.

Two major network meta-analyses published in 2020 found that balanced crystalloids were most advantageous for patients with septic shock (16, 17), a conclusion that contradicts the 2021 Sepsis Surviving Campaign Guidelines. Therefore, it is necessary to revisit the optimal fluid choice, incorporating the latest RCT findings.

## Research methods

This meta-analysis is conducted in accordance with the Preferred Reporting Items for Systematic Reviews and Meta-Analyses (PRISMA) guidelines and registered in the INPLASY database (Registration number 2024100049, DOI 10.37766).

### Data sources

**Figure 1:**
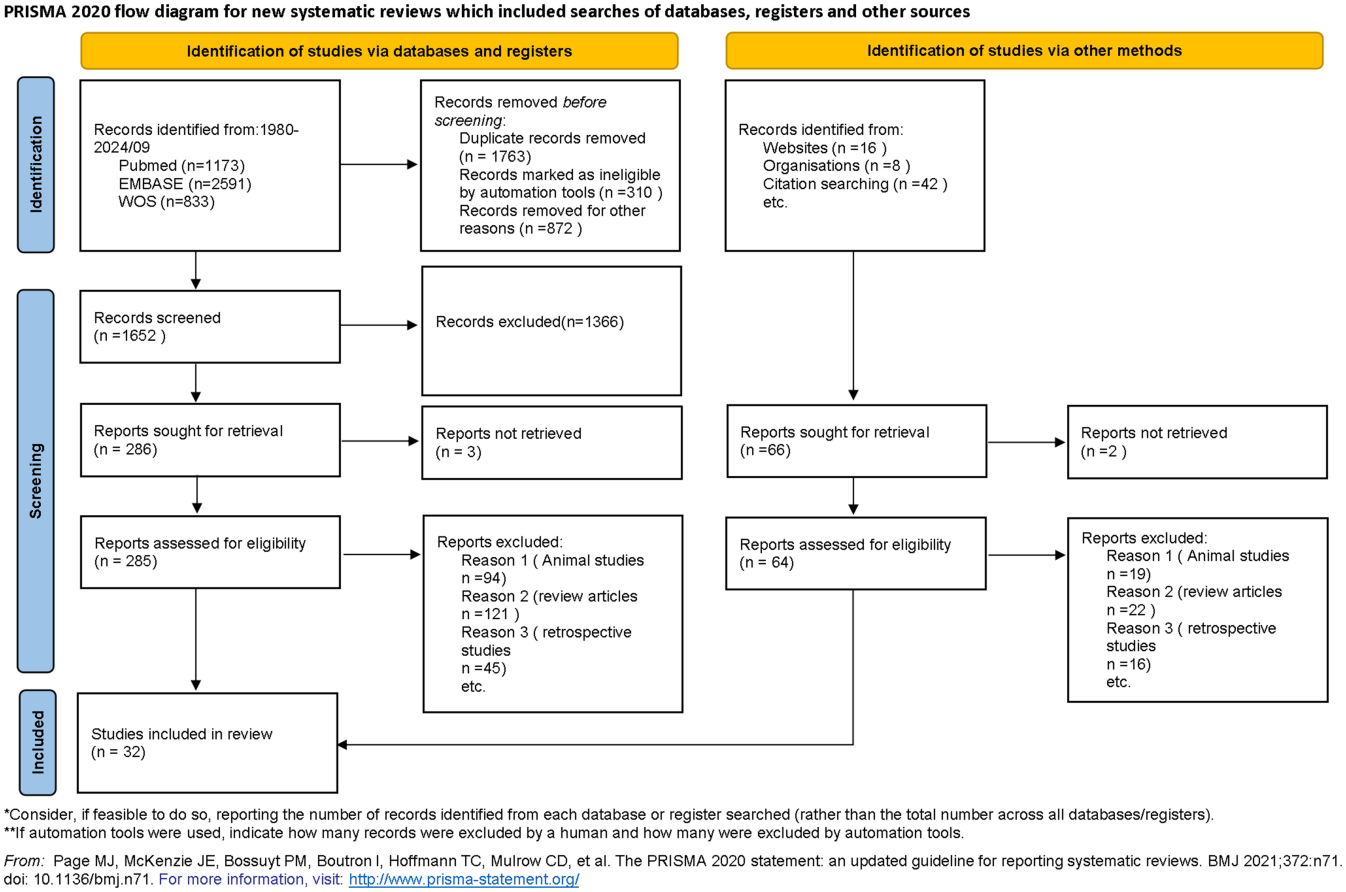
Flow chart of included literature. ^*^Consider, if feasible to do so, reporting the number of records identified from each database or register searched (rather than the total number across all databases/registers). ^**^If automation tools were used, indicate how many records were excluded by a human and how many were excluded by automation tools. From Page et al. (60). For more information, visit: http://www.prisma-statement.org/.

Following the PRISMA guidelines for systematic reviews and meta-analyses, a comprehensive literature search was conducted (18). Researchers systematically accessed databases such as PubMed, Embase, Web of Science, and the China Biology Medicine database (CBM), encompassing all pertinent literature up to September 1, 2024. Search terms included those related to sepsis, septic shock, and resuscitation fluids like saline, balanced solutions, and albumin. The search was restricted to articles in English and Chinese, and was limited to clinical trials. The detailed search strategy is presented in Figure 1, and additional information can be found in Supplementary material.

### Selection criteria

To ensure the relevance and quality of data, stringent inclusion criteria were established: Studies employing a randomized controlled trial (RCT) design; Studies with full texts available; Studies involving patients aged 16 years and older diagnosed with sepsis or infectious shock; Studies comparing the effects of different types of resuscitation fluids.

### Data extraction

Based on the established inclusion criteria, two researchers (Song Binglin and Liu Chun) independently extract data from studies that meet the requirements. The extracted data include basic information about the studies (such as the first author’s name and publication date), the number of patients, the types of resuscitation fluids used, mortality rates at various time points, incidence of AKI, and the need for RRT. Any discrepancies are resolved through discussion and consensus with a third researcher.

### Outcome measures

All-Cause Mortality: All-cause mortality refers to the proportion of deaths caused by any reason during the study period. For studies reporting outcomes at multiple time points, the longest observation period (e.g., during hospitalization, 30 days, or 90 days) was selected as the standard for mortality assessment. Acute Kidney Injury (AKI): Acute Kidney Injury (AKI) is defined as a rapid decline in kidney function over a short period, typically from several hours to a few days. The RIFLE criteria (Risk, Injury, Failure, Loss, and End-stage kidney disease) were used to assess the severity of renal dysfunction. According to the RIFLE criteria, AKI is classified into five levels based on changes in serum creatinine levels or urine output, with ‘Risk’ indicating mild damage and ‘End-stage’ representing end-stage renal disease. Rate of Renal Replacement Therapy (RRT): Renal Replacement Therapy (RRT) refers to artificial kidney support provided to patients with acute kidney injury through dialysis or filtration. The proportion of hospitalized patients requiring RRT was calculated. Length of Hospital Stay: Length of hospital stay refers to the total duration of hospitalization from admission to discharge, including both ICU and general ward stays. The duration of ICU stay and total hospital stay were analyzed separately, and the impact of different resuscitation fluids on hospital length of stay was also considered.

### Risk of bias assessment

**Figure 2:**
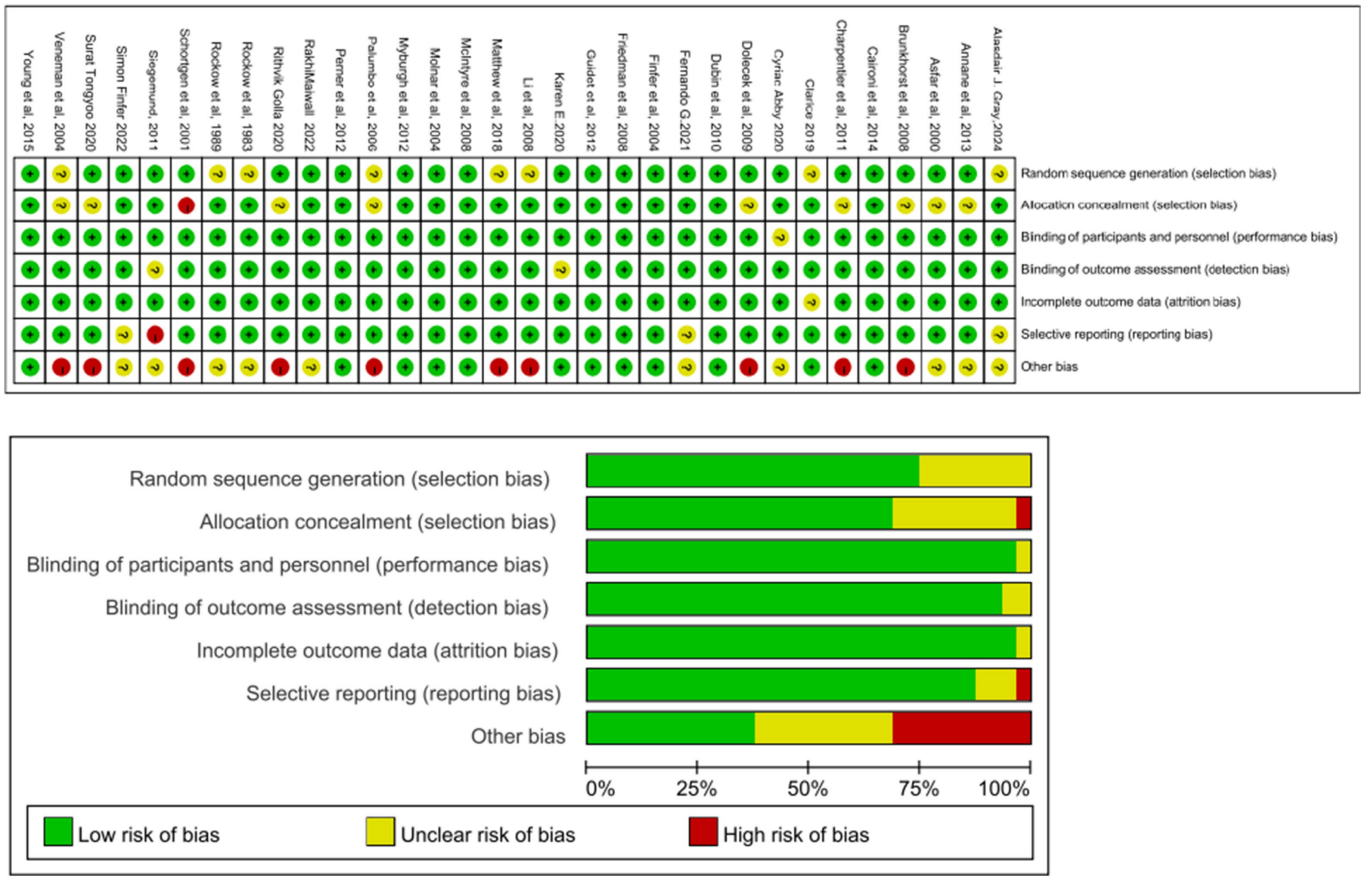
Assessment of the risk of bias for each study by the Cochrane collaboration tool.

To evaluate the quality of included studies, researchers use the Cochrane collaboration tool to assess the risk of bias for each study (19). The evaluation criteria include seven entries: randomization methods, concealment of allocation, blinding of researchers and participants, blinding of outcome assessors, data completeness, selective reporting of results, and other sources of bias. Each criterion is rated as “low risk,” “unclear,” or “high risk.” This assessment is independently performed by two researchers, and any disagreements are resolved through group discussion. Figure 2 illustrates this process.

### Data synthesis

Studies are categorized by the types of resuscitation fluids used: Interventions include two crystalloids [balanced crystalloids, comprising Lactated Ringer’s, Acetate Ringer’s, or PlasmaLytes, and saline (0.9% sodium chloride)], and five colloids [iso-oncotic albumin (4, 5%); hyper-oncotic albumin (20, 25%); low molecular weight hydroxyethyl starch (≤130kD, LHES); high molecular weight hydroxyethyl starch (≥200kD, H-HES); and gelatin].

### Statistical analysis

A network meta-analysis is conducted, using odds ratios for dichotomous data and mean differences for continuous data as effect size measures, both provided with 95% confidence intervals (20). When results are reported as medians and interquartile ranges (IQR) in multiple studies, their means and standard deviations are calculated based on sample size using calculators (21). A random-effects network meta-analysis model is used to synthesize effect sizes (22). This model accounts for variability in treatment effects across different studies and comparisons by considering the variance of random effects (i.e., heterogeneity variance). In network meta-analysis, it is initially assumed that the heterogeneity measure for all treatment comparisons is the same (23). Statistical assessments of inconsistency are performed in R (version 4.3.1) using the Rjags package (Martyn Plummer, Coventry, United Kingdom), and network plots are generated to identify relationships between different interventions. The model’s convergence is assessed by plotting Brooks-Gelman-Rubin diagnostic plots, trace plots, and density plots. Ranking probability plots are drawn, and the area under the cumulative ranking curve is calculated to identify the optimal intervention. The Surface Under the Cumulative Ranking Curve (SUCRA) and the mean ranks are analyzed. SUCRA values and rankograms are used to display the ranking of each intervention across different outcomes. The significance of SUCRA values lies in showing the percentage effectiveness achieved by each intervention under the hypothetical scenario of an ideal intervention with no uncertainty. Generally, a higher SUCRA value indicates better treatment efficacy.

### Patient or public involvement

Patients or the public were not involved in the design, conduct, reporting, or dissemination plans of our research.

## Results

A total of 32 randomized controlled trials (RCTs) conducted between 1983 and 2024 were included, involving 28,888 patients diagnosed with sepsis or septic shock (3, 7, 11, 13–15, 24–49). Among these, 15 were multicenter studies. The trials compared seven types of resuscitation fluids: balanced crystalloids (BC), saline, iso-oncotic albumin (Iso-Alb), hyper-oncotic albumin (Hyper-Alb), low molecular weight hydroxyethyl starch (L-HES), high molecular weight hydroxyethyl starch (HES), and gelatin.

The literature search identified 4,597 records, of which 352 full-text articles were assessed for eligibility. Finally, 25 trials were obtained from database searches and 7 from other sources, resulting in 32 RCTs meeting the inclusion criteria (Figure 1).

Risk of bias was evaluated using the Cochrane Collaboration tool, and the results are presented in Figure 2. This network meta-analysis focused on primary outcomes (all-cause mortality, acute kidney injury [AKI], and the need for continuous renal replacement therapy [CRRT]) and secondary outcomes (ICU length of stay and total hospital stay). Network diagrams of the comparisons are shown in Supplementary Figures 3, 6, 9, 12, with BC versus saline being the most common direct comparison. Heterogeneity forest plots, trace density plots, and convergence diagnostics are provided in Supplementary material.

### Mortality

**Figure 3:**
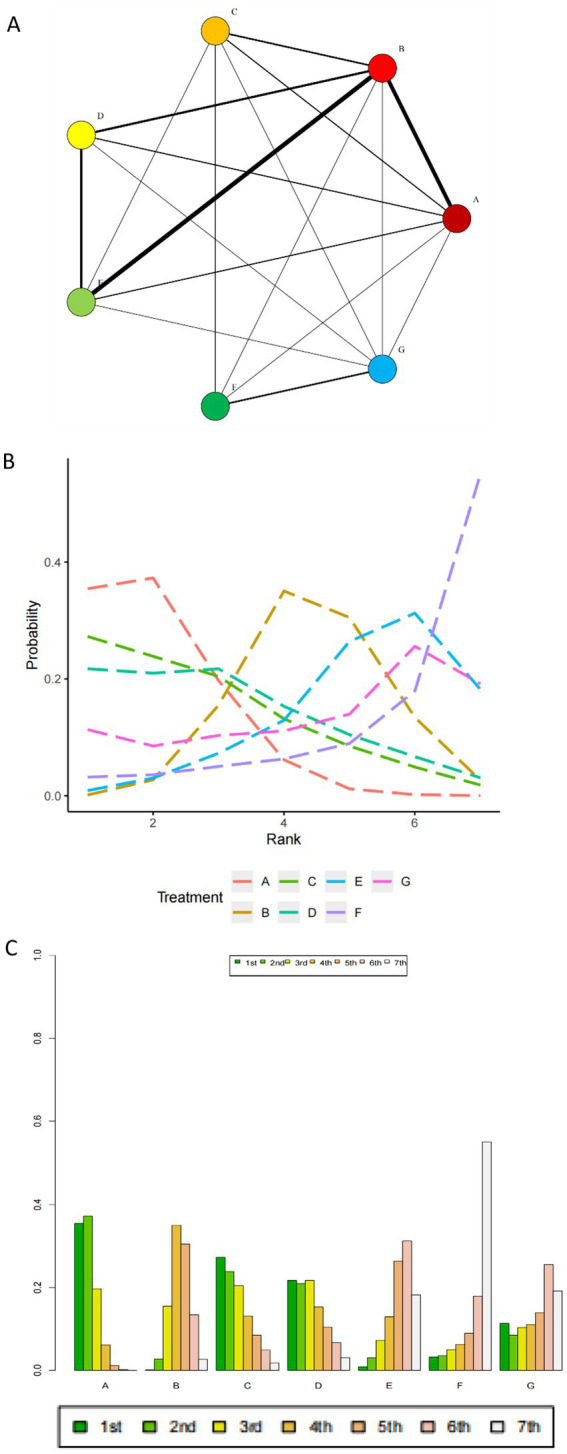
**(A)** Mortality network. [The network includes balanced crystalloids (BC), normal saline (Saline), iso-oncotic albumin (Iso-Alb), hyper-oncotic albumin (Hyper-Alb), low molecular weight hydroxyethyl starch (L-HES), high molecular weight hydroxyethyl starch (HES), and gelatin. Each node represents one of the resuscitation fluids, with lines connecting them indicating direct comparisons made in the included randomized controlled trials (RCTs). The thickness of the lines reflects the number of patients involved in each comparison, with thicker lines indicating more patients]. **(B)** SUCR plot of mortality. [This figure presents the Surface Under the Cumulative Ranking Curve (SUCRA) values for different resuscitation fluids with respect to all-cause mortality. The SUCRA values are plotted to rank the effectiveness of the fluids in reducing mortality, with balanced crystalloids (BC) showing the highest ranking. The fluids are ranked from highest to lowest based on their associated mortality rates: BC (83.1%), Iso-Alb (71.0%), Hyper-Alb (65.9%), Saline (42.6%), Gelatin (39.7%), L-HES (28.7%), and HES (18.6%)]. **(C)** Probability ranking plot. [32 eligible comparison networks. Before further research, all eligible studies were network screened and seven branches were selected, these included balanced crystalloid solution (BC), normal Saline (Saline), Iso-Alb, Hyper-Alb, L-HES, HES and Gelatin. The thickness of the lines represents the number of patients involved in the comparison. A, balanced crystalloid solution (BC); B, normal Saline (Saline); C, Iso-Alb; D, Hyper-Alb; E, L-HES; F, HES; G, Gelatin].

From 1983 to 2024, data on mortality from 32 RCTs involving 28,888 participants were analyzed (3, 7, 11, 13–15, 24–49). The mortality outcomes analyzed included in-hospital mortality, and mortality at 30 and 90 days. When multiple time points were reported in a study, the longest observation period was chosen for analysis. The Brooks-Gelman-Rubin diagnostic plots show that the median Potential Scale Reduction Factor (PSRF), after 50,000 iterations, tends toward 1 and stabilizes, indicating good model convergence and reliable analysis results, as detailed in Supplementary material. Table 1 presents the league tables for the primary outcomes. In terms of safety, balanced crystalloids (BC) are associated with a lower mortality rate compared to saline (0.9 [0.78, 0.99]). No significant differences were observed in patient survival rates among the other fluid medications studied. The mortality network for mortality is shown in Figures 3A–C present probability plots and treatment rankings based on SUCRA for mortality, showing the rankings of different resuscitation fluids in terms of all-cause mortality as follows, from lowest to highest mortality rates: Balanced Crystalloids A (83.1%), C (71.0%), D (65.9%), B (42.6%), G (39.7%), E (28.7%), and F (18.6%). [Where A, Balanced Crystalloids (BC); B, Saline; C, Iso-Oncotic Albumin (Iso-Alb); D, Hyper-Oncotic Albumin (Hyper-Alb); E, Low Molecular Weight Hydroxyethyl Starch (L-HES); F, High Molecular Weight Hydroxyethyl Starch (HES); G, Gelatin.] Heterogeneity forest plots, trace density plots, and convergence diagnostic plots are also available in Supplementary material.

**Table 1:** Comparison of mortality between different fluid types (odds ratios with 95% confidence intervals).

A						
0.9 (0.78, 0.99)	B					
0.98 (0.78, 1.19)	1.09 (0.89, 1.32)	C				
0.96 (0.76, 1.17)	1.07 (0.88, 1.29)	0.98 (0.75, 1.29)	D			
0.85 (0.7, 1.01)	0.95 (0.81, 1.12)	0.87 (0.68, 1.12)	0.89 (0.7, 1.13)	E		
0.77 (0.56, 1.07)	0.87 (0.63, 1.21)	0.79 (0.56, 1.15)	0.81 (0.56, 1.19)	0.91 (0.64, 1.3)	F	
0.86 (0.63, 1.18)	0.97 (0.71, 1.32)	0.89 (0.64, 1.24)	0.9 (0.64, 1.29)	1.02 (0.73, 1.41)	1.12 (0.75, 1.65)	G

Data are odds ratios (95% confidence intervals) for column-defined treatment versus row-defined treatment. With respect to safety, an odds ratio greater than 1 favored the defined treatment. (The 95% confidence interval was 1 as the null value.) Statistically significant results are marked with yellow filling. A, Balanced Crystalloid Solution (BC); B, Normal Saline (Saline); C, Iso-Alb; D, Hyper-Alb; E, L-HES; F, HES; G, Gelatin.

### Acute kidney injury

In studies on acute kidney injury (AKI), 15 trials involving 13,742 participants reported data on AKI, with network meta-analysis results illustrated in Table 2. In terms of safety, balanced crystalloids (BC), saline (Saline), and gelatin (Gelatin) are associated with a lower incidence of AKI compared to high molecular weight hydroxyethyl starch (HES) [0.54 (0.34, 0.84), 0.57 (0.35, 0.92), 2.37 (1.06, 5.45)]. Additionally, balanced crystalloids (BC) demonstrate a significantly lower incidence of AKI compared to low molecular weight hydroxyethyl starch (L-HES) [0.78 (0.58, 0.99)]. No significant differences were observed in AKI incidence among the other studied fluid medications. Supplementary Figures 7, 8 present probability plots and treatment rankings based on SUCRA for AKI incidence, ranking the different resuscitation fluids as follows from lowest to highest AKI incidence: Gelatin (G, 80.7%), Balanced Crystalloids (A, 76.8%), Saline (B, 62.2%), Iso-Oncotic Albumin (C, 48.6%), Low Molecular Weight Hydroxyethyl Starch (E, 28.3%), High Molecular Weight Hydroxyethyl Starch (F, 3%).

**Table 2:** Comparison of AKI incidence between different fluid types (odds ratios with 95% confidence intervals).

A					
0.94 (0.77, 1.11)	B				
0.87 (0.6, 1.22)	0.93 (0.66, 1.29)	C			
0.78 (0.58, 0.99)	0.82 (0.64, 1.04)	0.89 (0.59, 1.32)	E		
0.54 (0.34, 0.84)	0.57 (0.35, 0.92)	0.61 (0.35, 1.11)	0.69 (0.42, 1.18)	F	
1.27 (0.5, 3.27)	1.35 (0.53, 3.53)	1.46 (0.55, 4.01)	1.64 (0.63, 4.41)	2.37 (1.06, 5.45)	G

Data are odds ratios (95% confidence intervals) for column-defined treatment versus row-defined treatment. With respect to safety, an odds ratio greater than 1 favored defined treatment. (The 95% confidence interval was 1 as the null value.) Significant results are marked with yellow filling.

### Renal replacement therapy

In studies on renal replacement therapy (RRT), 17 trials comprising 16,515 participants reported data on RRT, with the main outcomes of the network meta-analysis shown in Table 3. Compared to high molecular weight hydroxyethyl starch (HES), balanced crystalloids (BC), saline (Saline), iso-oncotic albumin (Iso-Alb), hyper-oncotic albumin (Hyper-Alb), and low molecular weight hydroxyethyl starch (L-HES) are associated with a lower occurrence of renal replacement events [0.48 (0.3, 0.72), 0.49 (0.31, 0.76), 0.49 (0.28, 0.81), 0.27 (0.1, 0.69), 0.61 (0.36, 0.99)]. Additionally, balanced crystalloids (BC) show a marginally significant reduction in renal replacement events compared to low molecular weight hydroxyethyl starch (L-HES) [0.79 (0.59, 1.03)]. There were no significant differences in the rates of CRRT among the other fluid medications studied. Supplementary Figures 10, 11 display probability plots and treatment rankings based on SUCRA for RRT incidence, with the rankings from lowest to highest RRT incidence as follows: Hyper-Oncotic Albumin (D, 94.1%), Balanced Crystalloids (A, 67.5%), Iso-Oncotic Albumin (C, 62.1%), Saline (B, 61.1%), Gelatin (G, 34.3%), Low Molecular Weight Hydroxyethyl Starch (E, 29.1%), and High Molecular Weight Hydroxyethyl Starch (F, 1.4%). Heterogeneity forest plots, trace density plots, and convergence diagnostic plots are provided in Supplementary material.

**Table 3:** Comparison of RRT (Renal Replacement Therapy) incidence between different fluid types (odds ratios with 95% confidence intervals).

A						
0.97 (0.8, 1.18)	B					
0.98 (0.68, 1.4)	1 (0.72, 1.42)	C				
1.78 (0.77, 4.13)	1.83 (0.78, 4.36)	1.83 (0.73, 4.56)	D			
0.79 (0.59, 1.03)	0.81 (0.62, 1.04)	0.81 (0.53, 1.21)	0.44 (0.18, 1.07)	E		
0.48 (0.3, 0.72)	0.49 (0.31, 0.76)	0.49 (0.28, 0.81)	0.27 (0.1, 0.69)	0.61 (0.36, 0.99)	F	
0.77 (0.43, 1.37)	0.79 (0.45, 1.42)	0.79 (0.46, 1.35)	0.43 (0.15, 1.2)	0.98 (0.53, 1.83)	1.6 (0.91, 2.95)	G

Data are odds ratios (95% confidence intervals) for column-defined treatment versus row-defined treatment. With respect to safety, an odds ratio greater than 1 favored defined treatment. (The 95% confidence interval was 1 as the null value.) Significant results are marked with yellow filling.

### ICU length of stay

Nine trials involving 16,184 participants reported data related to the ICU length of stay, expressed as the mean difference (MD) with a 95% confidence interval (CI) for this continuous variable. Outcome measures reported as medians and interquartile ranges (IQR) were converted into means and standard deviations based on sample size using a calculator (50). Data conversions were performed in the R statistical package, using the standardized mean difference (SMD) as the effect size and zero as the null effect value. The league table results (Table 4) were statistically significant, ranking the ICU stay duration from shortest to longest as follows: A (Balanced Crystalloids—BC), F (High Molecular Weight Hydroxyethyl Starch have provided new evidence regarding the effectiveness of HES), C (Iso-Oncotic Albumin—Iso-Alb), B (Saline), D (Hyper-Oncotic Albumin—Hyper-Alb), E (Low Molecular Weight Hydroxyethyl Starch—L-HES). The Brooks-Gelman-Rubin diagnostic plots demonstrate that the median Potential Scale Reduction Factor (PSRF) stabilized at 1 after 50,000 iterations, indicating good model convergence and reliable analysis results (see Supplementary material). The heterogeneity forest plots, trace density plots, and convergence diagnostic plots are also provided in Supplementary material.

**Table 4:** Comparison of ICU length of stay between different fluid types (odds ratios with 95% confidence intervals).

A					
0.85 (0.3, 2.36)	B				
0.88 (0.19, 4.12)	1.05 (0.22, 4.85)	C			
0.76 (0.16, 3.59)	0.9 (0.19, 4.23)	0.86 (0.11, 6.79)	D		
0.35 (0.06, 2.11)	0.41 (0.1, 1.8)	0.4 (0.05, 3.33)	0.46 (0.06, 3.9)	E	
0.99 (0.09, 10.52)	1.18 (0.14, 9.79)	1.13 (0.08, 15.47)	1.31 (0.09, 17.96)	2.82 (0.21, 36.73)	F

Data are odds ratios (95% confidence intervals) for column-defined treatment vs. row-defined treatment. For duration, an odds ratio greater than 1 favored defining treatment. (The 95% confidence interval was 0 as the null value.) Significant results are marked with yellow filling.

### Hospital length of stay

Nine trials comprising 16,184 participants also reported data on the total hospital length of stay, expressed as the mean difference (MD) with a 95% confidence interval (CI). The league table results (Table 5) demonstrated statistical significance, ordering the length of hospital stay from shortest to longest as follows: A (Balanced Crystalloids—BC), B (Saline), D (Hyper-Oncotic Albumin—Hyper-Alb), C (Iso-Oncotic Albumin—Iso-Alb), E (Low Molecular Weight Hydroxyethyl Starch—L-HES), F (High Molecular Weight Hydroxyethyl Starch—HES). Brooks-Gelman-Rubin diagnostic plots show that both the median and the 97.5% percentile of the PSRF approached 1 after 50,000 iterations, suggesting good model convergence and reliable results (see Supplementary material). Table 5 presents the league table for the main outcomes, and additional diagnostic visualizations are included in Supplementary material.

**Table 5:** Comparison of total hospital days between different fluid types (odds ratios with 95% confidence intervals).

A					
0.92 (0.62, 1.35)	B				
0.81 (0.45, 1.44)	0.88 (0.5, 1.57)	C			
0.84 (0.45, 1.51)	0.91 (0.51, 1.63)	1.03 (0.47, 2.23)	D		
0.69 (0.36, 1.36)	0.75 (0.44, 1.32)	0.84 (0.39, 1.91)	0.82 (0.38, 1.87)	E	
0.74 (0.26, 2.08)	0.81 (0.31, 2.1)	0.91 (0.3, 2.78)	0.88 (0.29, 2.72)	1.08 (0.35, 3.18)	F

Data are odds ratios (95% confidence intervals) for column-defined treatment vs. row-defined treatment. For duration, an odds ratio greater than 1 favored defining treatment. (The 95% confidence interval was 0 as the null value.) Significant results are marked with yellow filling. A, Balanced Crystalloid Solution (BC); B, Normal Saline (Saline); C, Iso-Alb; D, Hyper-Alb; E, L-HES; F, HES; G, Gelatin.

## Discussion

This systematic review and network meta-analysis (NMA) integrates both direct and indirect evidence, providing a comprehensive summary of the effects of resuscitation fluids on five outcomes in patients with septic shock, including mortality and the incidence of AKI. Our study demonstrates that balanced crystalloids (BC) are associated with a lower mortality rate compared to normal saline (0.9 [0.78, 0.99]). When compared with other resuscitation fluids in the study, there was no significant difference in patient survival rates. However, balanced crystalloids (BC) exhibited the highest SUCRA value (83.1%) and were also associated with the shortest ICU length of stay and total hospital stay. Gelatin showed the greatest advantage in terms of kidney injury, with the highest SUCRA value (80.7%). Hyper-oncotic albumin exhibited the lowest incidence of renal replacement therapy (RRT) events, showing the highest SUCRA value (94.1%). Therefore, it is not reasonable to treat all crystalloids as homogeneous; more emphasis should be placed on selecting balanced crystalloids for septic patients.

Iso-oncotic albumin solutions have an osmolarity close to that of plasma, while hyper-oncotic albumin solutions have relatively higher osmolarity (51). Iso-oncotic albumin is primarily used for volume resuscitation and effectively retains fluid, whereas hyper-oncotic albumin helps maintain target plasma albumin levels and mobilizes endogenous fluid to stabilize effective circulating volume (52). Our study found that hyper-oncotic albumin (Hyper-Alb) is most advantageous in reducing the need for renal replacement therapy compared to other fluids. Despite requiring the least amount of fluid to achieve resuscitation targets, its high cost limits its feasibility as the preferred resuscitation fluid. Although high-molecular-weight hydroxyethyl starch (HES) has been withdrawn from the market, low-molecular-weight HES is still widely used in surgical and trauma patients (41). However, our study showed that, compared to balanced crystalloids, low-molecular-weight HES was associated with higher mortality, increased kidney injury risk, and longer hospital stays. In a 2023 review by Timo Mayerhöfer, it was pointed out that the use of gelatin might trigger severe allergic reactions, possibly related to allergies to galactose-*α*-1,3-galactose (53). Additionally, gelatin may increase the risk of bleeding, CRRT events, and death. Our network meta-analysis did not find any advantages of gelatin compared to other resuscitation fluids, so its use in resuscitation, especially in septic patients, is not recommended.

In a 2014 network meta-analysis by Bram, it was found that in sepsis patients, the use of balanced crystalloids or albumin for resuscitation appeared to be associated with reduced mortality compared to other fluids. A large NMA by Chien (16) indicated that balanced crystalloids and albumin were more effective than L-HES and normal saline in reducing mortality in septic patients. Similarly, Li’s (17) study also recommended balanced solutions (BS) as the first-line resuscitation fluid for infectious shock. Our study reached similar conclusions regarding the advantages of BS, but with the inclusion of the latest large-scale RCTs, we found that balanced crystalloids (BC) had the highest SUCRA value for mortality reduction. Gelatin, however, had worse outcomes in terms of mortality, continuous renal replacement therapy (CRRT), and hospital stay duration, which contrasts with the conclusions of previous network meta-analyses.

Among all the available literature, this is the third article to specifically perform a network meta-analysis of fluid resuscitation in septic shock. Compared to previous studies, we not only ranked fluids based on SUCRA values but also compared the results using multi-node NMA. We included the latest relevant data, incorporating the largest number of patients. Furthermore, we not only reassessed major outcomes like mortality and kidney injury but also explored hospital length of stay, a factor less frequently addressed in previous studies, leading to meaningful conclusions. Our findings can guide clinicians in fluid selection and may provide evidence for future guideline updates.

However, regardless of the type of crystalloid, large fluid volumes are required to achieve resuscitation goals, which raises concerns about the potential risks of fluid overload (54, 55). Studies have shown that intravenous fluids aimed at restoring organ perfusion can damage vascular integrity and lead to organ dysfunction (56). Observational studies indicate that large-volume fluid resuscitation is associated with increased mortality, but these studies may be influenced by unmeasured variables (e.g., more fluids given to patients with more severe conditions) (55, 57). Due to insufficient evidence, the 2021 Surviving Sepsis Campaign guidelines did not recommend restrictive or liberal fluid therapy within the first 24 h of resuscitation for septic and septic shock patients. However, with increasing attention to “protective hemodynamics,” a meta-analysis and review published in 2025 found that mortality was lower in the low blood pressure target group (58, 59). This may further provide new evidence for exploring restrictive fluid resuscitation.

### Limitations

First, in the sepsis trials involving 28,888 samples, most were two-arm trials including only randomized controlled trials (RCTs). Data on colloids was scarce and statistically insignificant, so no clear ranking could be derived. Additionally, many studies used quartile data for continuous variables, which we converted, potentially introducing bias into the results. Finally, our study has thus far focused only on fluid types and has not addressed the specific volumes of fluid used during resuscitation, leaving room for further investigation into the relationship between different fluid volumes and outcomes.

## Conclusion

In septic patients, balanced crystalloids show superiority over other resuscitation fluids concerning survival, renal protection, and hospital stay lengths.

## Data Availability

The datasets presented in this study can be found in online repositories. The names of the repository/repositories and accession number(s) can be found in the article/Supplementary material.
