# Supplementary material for: Fluid resuscitation in adults with severe infection and sepsis: a systematic review and network meta-analysis
Source: Front Med (Lausanne). 2025 Jun 17;12:1543586. doi: 10.3389/fmed.2025.1543586 (PMC12209188; doi:10.3389/fmed.2025.1543586)
Supplement: Supplementary file 1 [file Data_Sheet_1.docx]

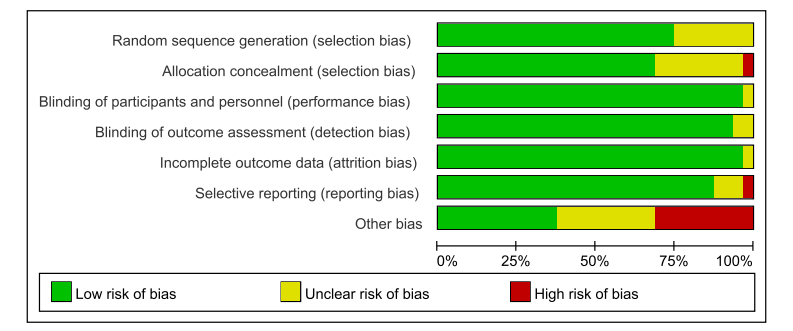

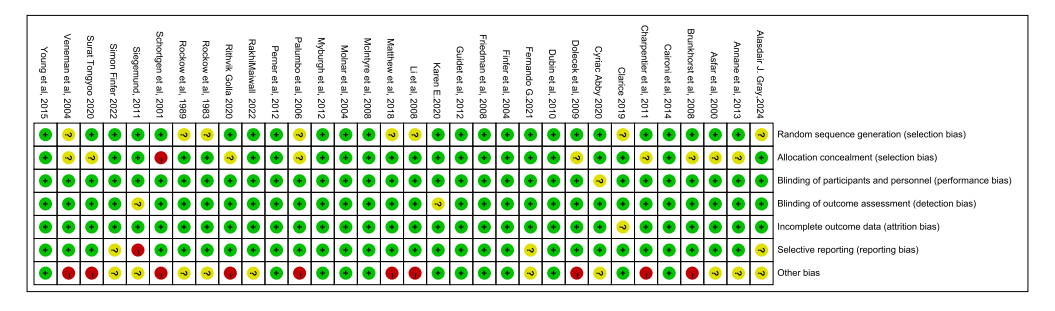

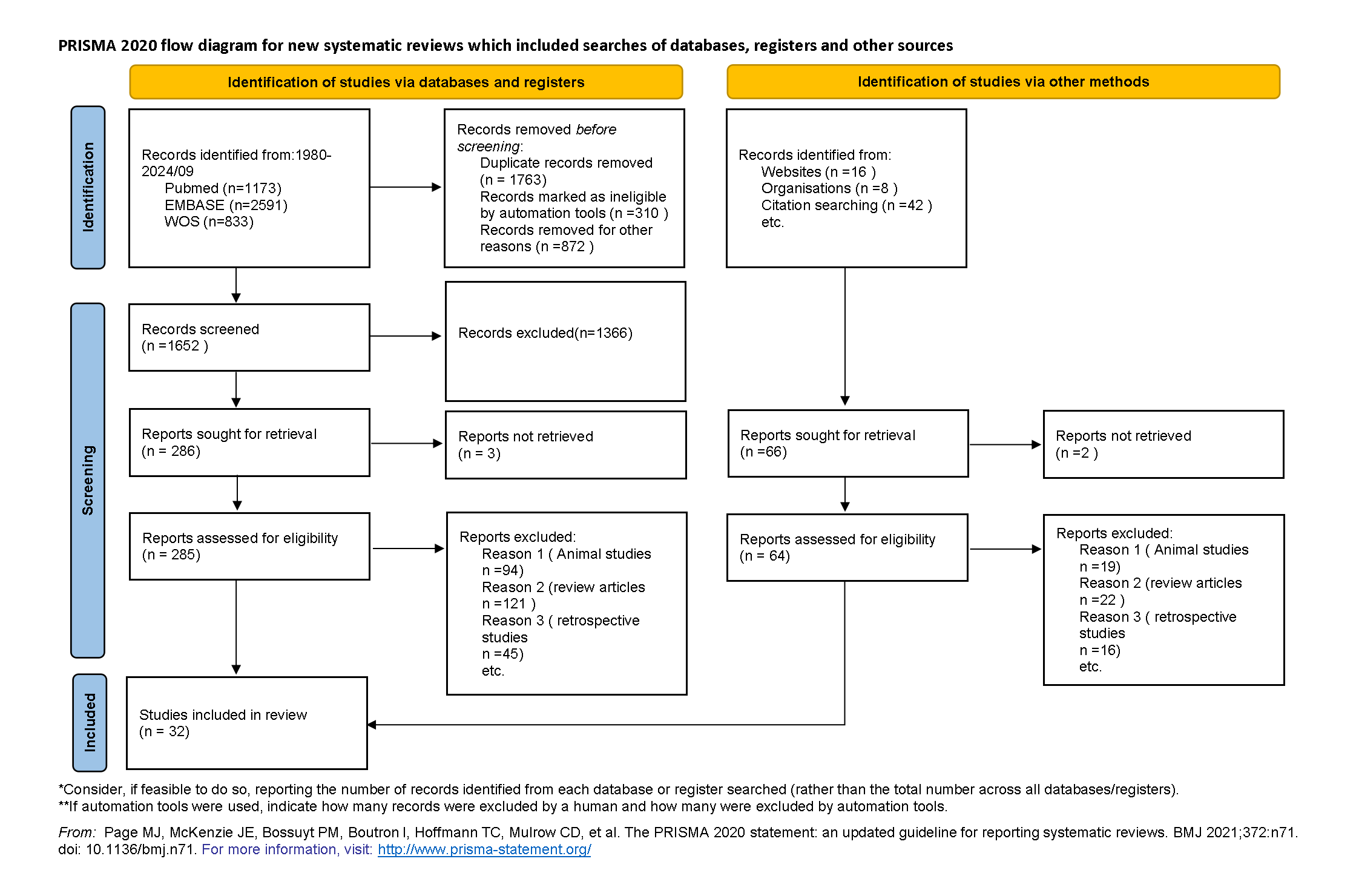
 Supplemental Figure 1：Flow chart of included literature

Supplemental Figure 2: Assessment of the risk of bias for each study by the Cochrane Collaboration tool


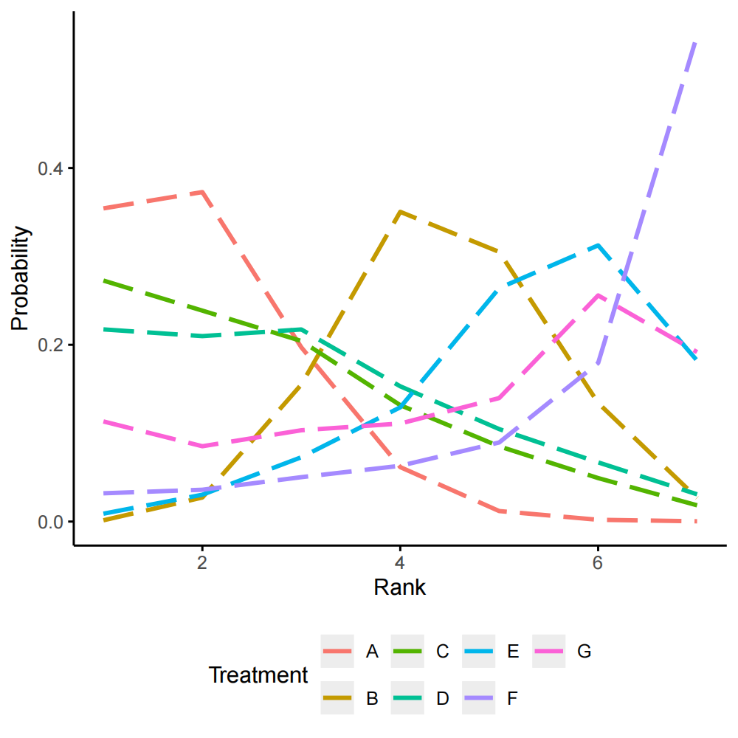

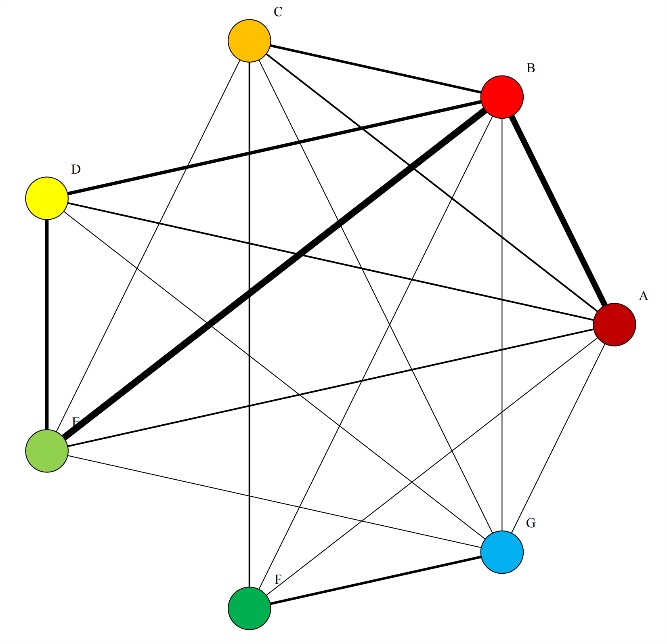


Supplemental Figure 3: Mortality network Supplemental Figure 4: SUCR plot of mortality


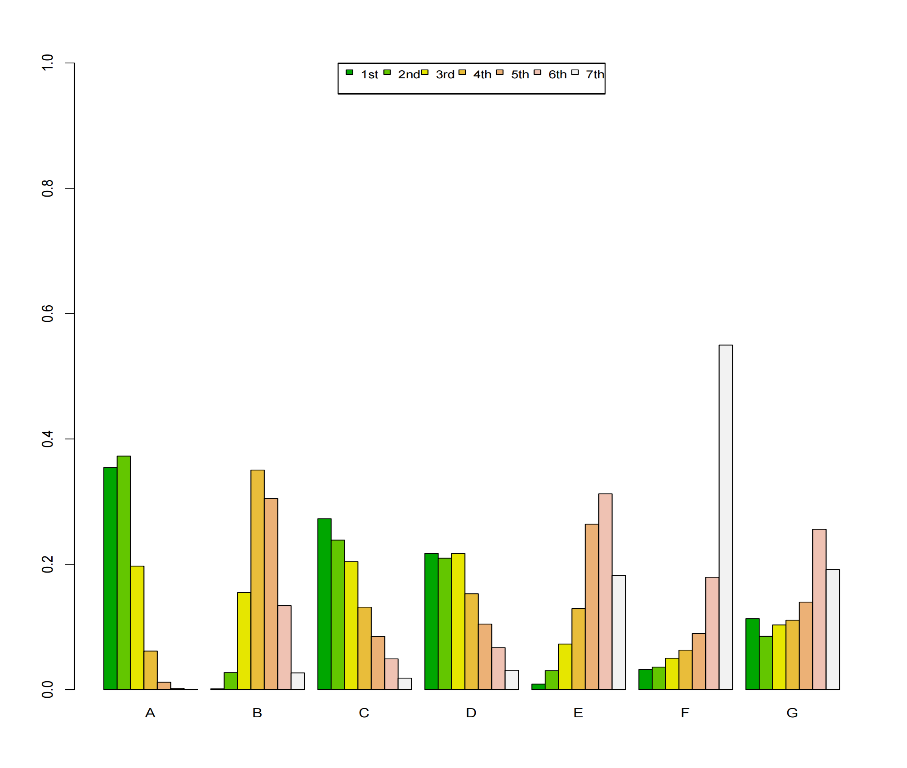


Notes: 32 eligible comparison networks. Before further research, all eligible studies were network screened and seven branches were selected, These included balanced crystalloid solution (BC), normal Saline (Saline), Iso-Alb, Hyper-Alb, L-HES, HES and Gelatin. The thickness of the lines represents the number of patients involved in the comparison. A= balanced crystalloid solution (BC), B= normal Saline (Saline), C= Iso-Alb, D= Hyper-Alb, E= L-HES, F= HES, G= Gelatin.


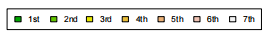


Supplemental Figure 5: Probability ranking plot


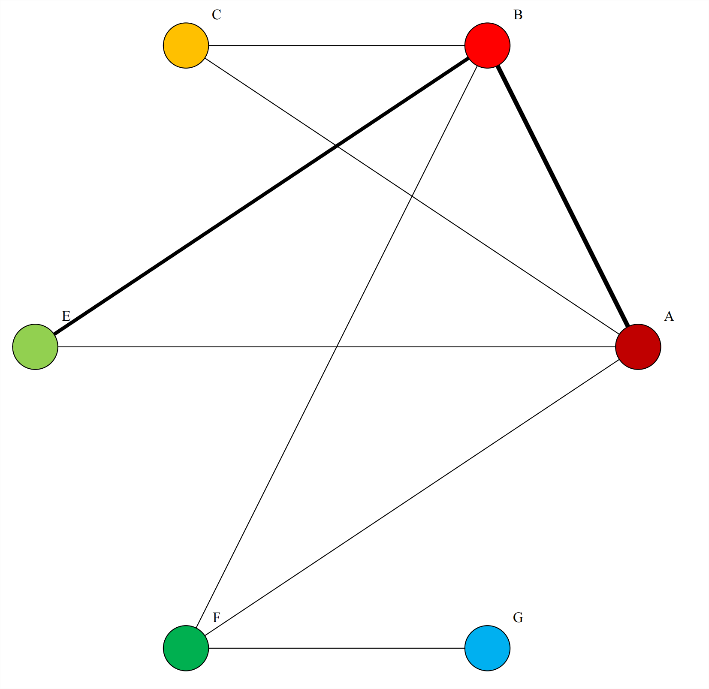

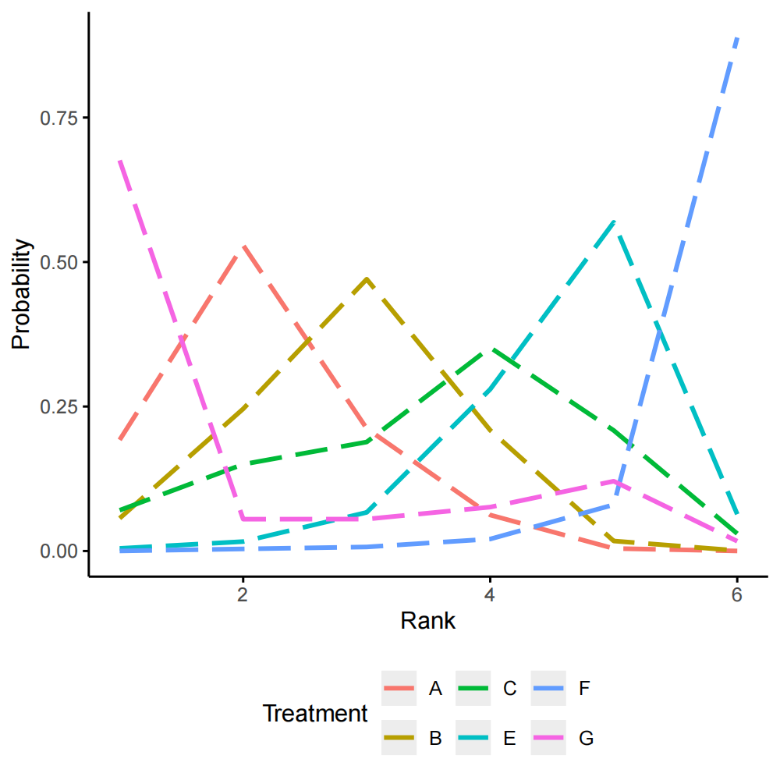
Supplemental Figure 6: AKI network diagram Supplemental Figure 7: Curve of AKISUCRA


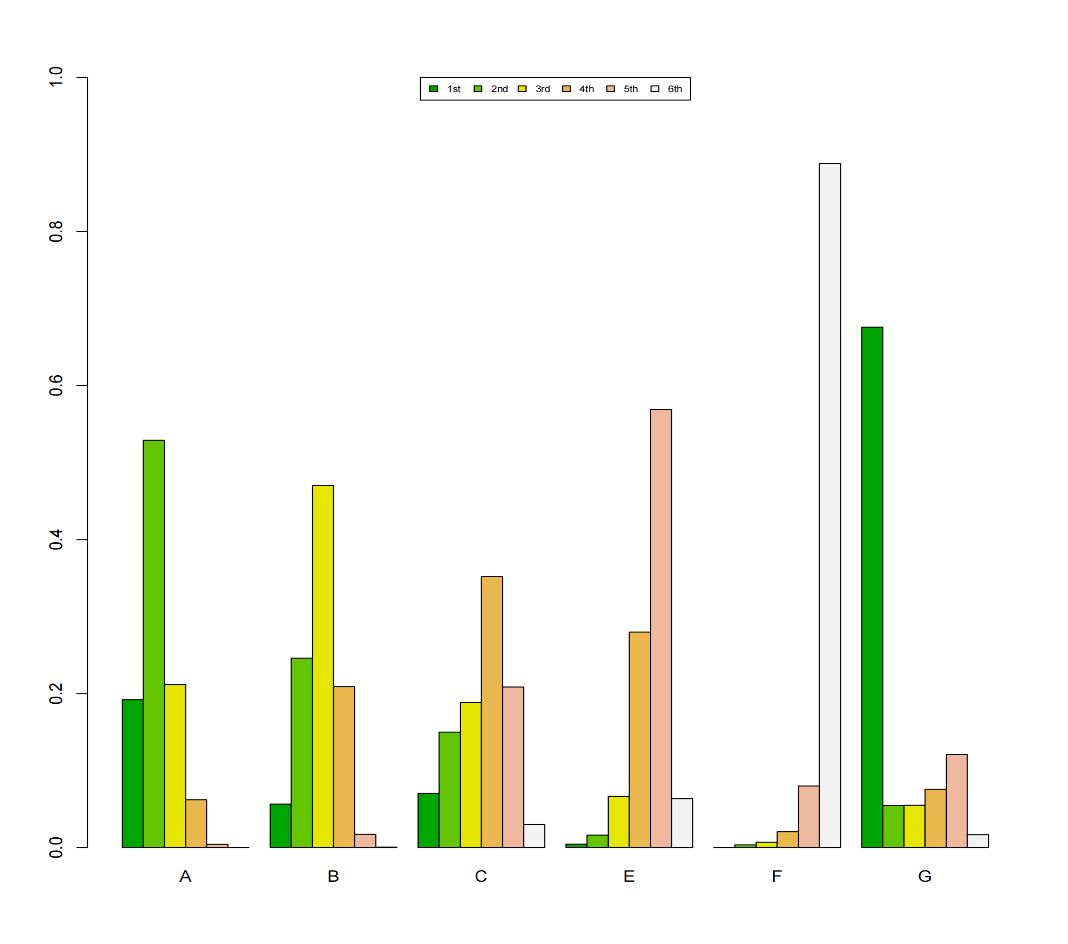


Nnotes: 15 eligible comparison networks. Before further study, all eligible studies were network screened and six branches were selected, including balanced crystalloid (BC), Saline (Saline), Iso-Alb, low molecular weight hydroxyethyl starch (L-HES), high molecular weight hydroxyethyl starch (HES), and Gelatin.


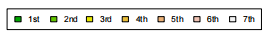


Supplemental Figure 8: AKI probability ranking diagram


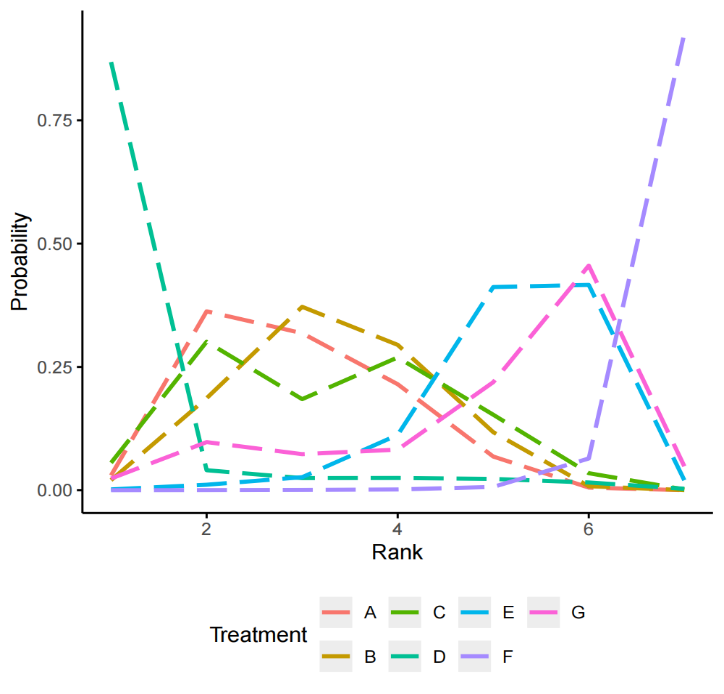

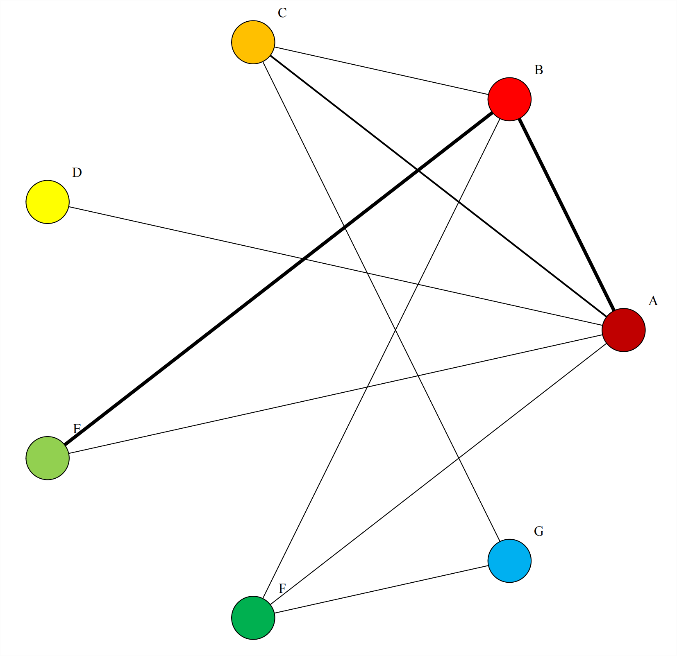


Supplemental Figure 9: CRRT network diagram Supplemental Figure 10: CRRTSUCRA graph


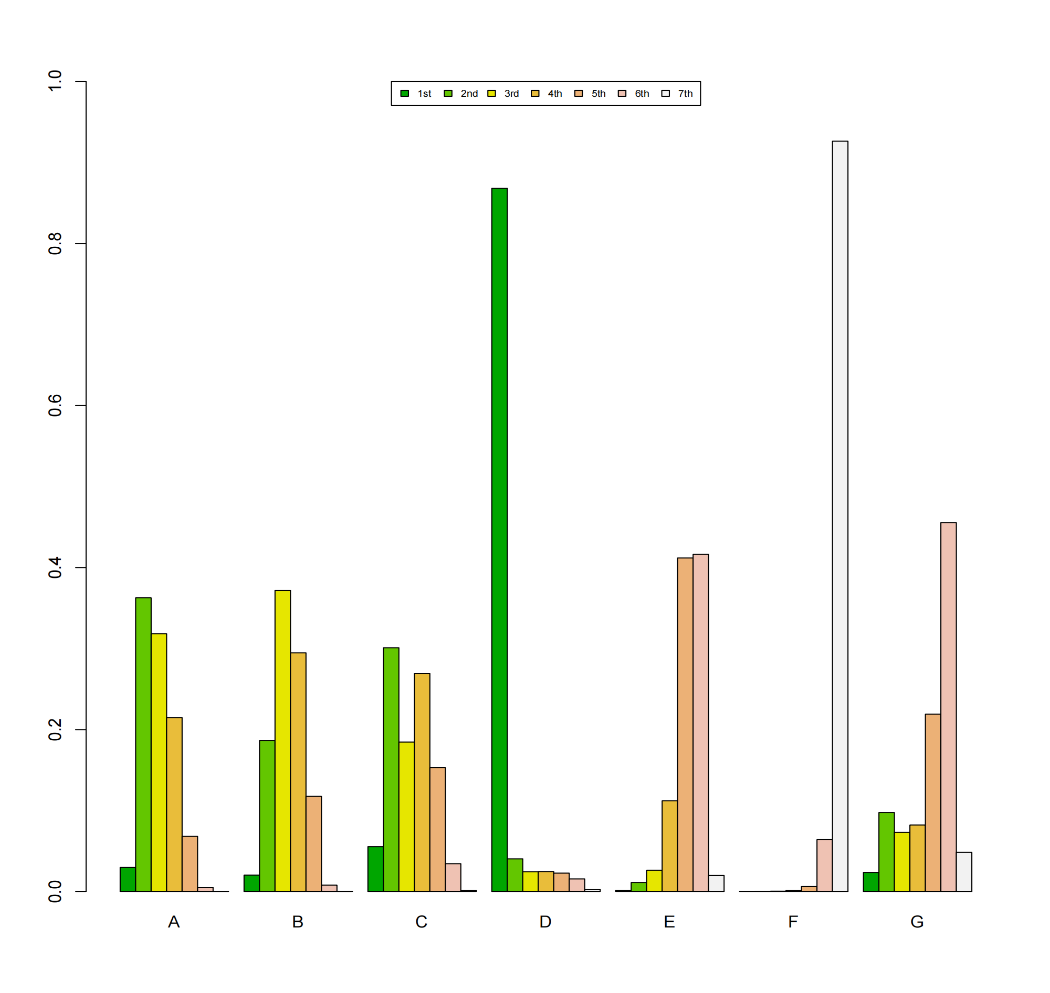


Notes: 17 eligible comparison networks. Before further research, all eligible studies were network screened and seven branches were selected, These included balanced crystalloid solution (BC), normal Saline (Saline), Iso-Alb, Hyper-Alb, L-HES, HES and Gelatin.


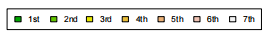


Supplemental Figure 11: CRRT probability ranking


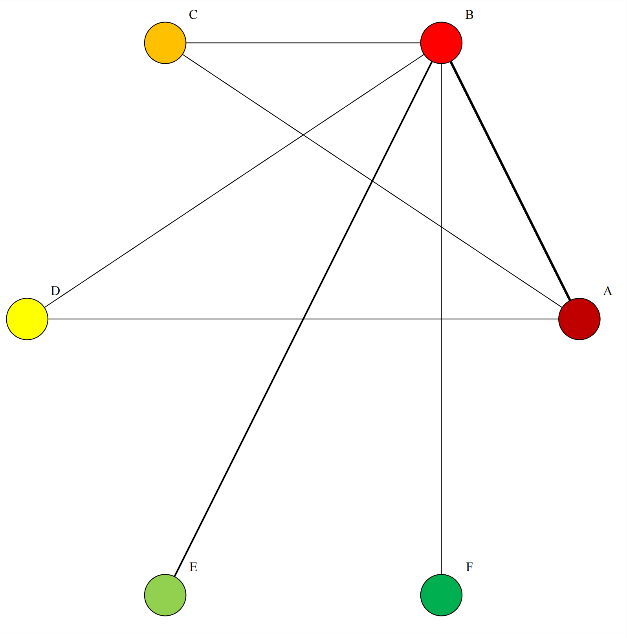

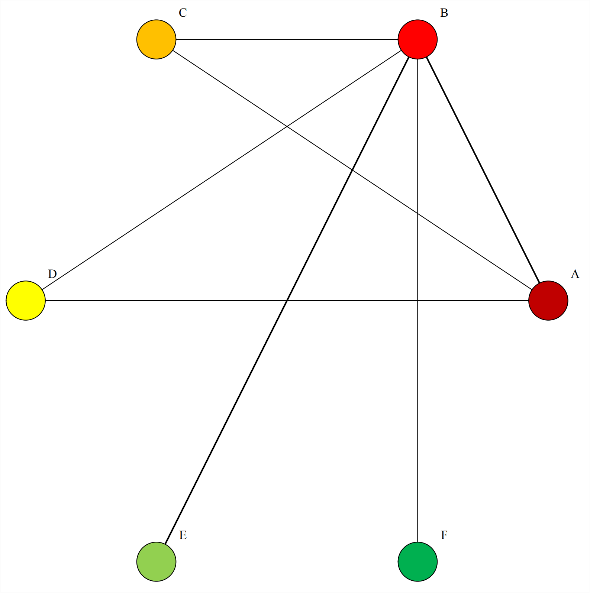


Supplemental Figure 12: ICU length of stay and total length of stay: nine eligible comparison networks. Six branches were selected, including balanced crystalloid solution (BC), normal Saline (Saline), Iso-Alb, Hyper-Alb, low molecular weight hydroxyethyl starch (L-HES) and high molecular weight hydroxyethyl starch (HES).

A= balanced crystalloid solution (BC), B= normal Saline (Saline), C= Iso-Alb, D= Hyper-Alb, E= L-HES, F= HES, G= Gelatin.
