# Supplementary material for: Fluid resuscitation in adults with severe infection and sepsis: a systematic review and network meta-analysis
Source: Front Med (Lausanne). 2025 Jun 17;12:1543586. doi: 10.3389/fmed.2025.1543586 (PMC12209188; doi:10.3389/fmed.2025.1543586)
Supplement: Supplementary file 2 [file Data_Sheet_2.doc]

Table A ：Included Studies

| **ID** | **Study Name** | **Year** | **Setting** | **No.of patients** | **Drug name1** | **Drug name2** | **Drug name3、4、5** |
| --- | --- | --- | --- | --- | --- | --- | --- |
| 1 | Rockow et al, | 1983 | single centre | 25(11/7/7) | Saline | IsoAlb | L-HES |
| 2 | Rockow et al, | 1989 | single centre | 20(10/10) | Iso-Alb | H-HES |  |
| 3 | Asfar et al, | 2000 | single centre | 34(16/18) | H-HES | Gelatin |  |
| 4 | Schortgen et al, | 2001 | single centre | 129(65/64) | H-HES | Gelatin |  |
| 5 | Finfer et al, | 2004 | multicentre | 1218(615/606) | Saline | IsoAlb |  |
| 6 | Molnar et al, | 2004 | single centre | 30(15/15) | H-HES | Gelatin |  |
| 7 | Veneman et al, | 2004 | single centre | 61(16/15/30) | Saline | Hyper-Alb | L-HES |
| 8 | Palumbo et al, | 2006 | single centre | 20(10/10) | Hyper-Alb | L-HES |  |
| 9 | Brunkhorst et al, | 2008 | multicentre | 535(274/261) | BC | H-HES |  |
| 10 | Friedman et al, | 2008 | single centre | 42(15/27) | 4%Iso-Alb | H-HES |  |
| 11 | Li et al, | 2008 | single centre | 30(15/15) | Saline | L-HES |  |
| 12 | McIntyre et al, | 2008 | single centre | 40(19/21) | Saline | H-HES |  |
| 13 | Dolecek et al, | 2009 | *single centre* | 56(30/26) | Hyper-Alb | L-HES |  |
| 14 | Dubin et al, | 2010 | single centre | 25(13/12) | Saline | L-HES |  |
| 15 | Charpentier et al, | 2011 | multicentre | 792(393/399) | Saline | Hyper-Alb |  |
| 16 | Siegemund, | 2011 | single centre | 241(124/117) | Hyper-Alb | L-HES |  |
| 17 | Guidet et al, | 2012 | single centre | 194(99/95) | L-HES | Saline |  |
| 18 | Myburgh et al, | 2012 | multicentre | 1921(976/945) | L-HES | Saline |  |
| 19 | Perner et al, | 2012 | multicentre | 798(400/398) | BC | L-HES |  |
| 20 | Annane et al, | 2013 | multicentre | 1180(37/557/59/375/152) | BC | Saline | Hyper-Alb、L-HES、Gelatin |
| 21 | Caironi et al, | 2014 | multicentre | 1781(893/888) | Saline | 20%Hyper-Alb |  |
| 22 | Young et al, | 2015 | single centre | 77(35/42) | BC | Saline |  |
| 23 | Matthew et al, | 2018 | multicentre | 5381(2735/2646) | BC | Saline |  |
| 24 | Alasdair J. Gray, | 2024 | multicentre | 296(149/147) | BC | Iso-Alb |  |
| 25 | Rithvik Golla | 2020 | multicentre | 160(23/32) | BC | Saline |  |
| 26 | RakhiMaiwall | 2022 | *single centre* | *100(50/50)* | BC | Hyper-Alb |  |
| 27 | Surat Tongyoo | 2020 | multicentre | 206(104/102) | Iso-Alb | Gelatin |  |
| 28 | Karen E | .2020 | multicentre | 367(142/225) | BC | Saline |  |
| 29 | Clarice | 2019 | single centre | 360(180/180) | BC | Hyper-Alb |  |
| 30 | Cyriac Abby | 2020 | multicentre | 448(248/200) | Saline | Iso-Alb |  |
| 31 | Fernando G. | 2021 | multicentre | 10520(5230/5290) | BC | Hyper-Alb |  |
| 32 | Simon Finfer | 2022 | multicentre | 5037(2515/2522) | phenylephrine | norepinephrine |  |

Table B：The basic information of the literature was included

| **Author, Year** | **Country** | **Centers** | **Type of liquid** | **Primary outcome** | **Mortality** |
| --- | --- | --- | --- | --- | --- |
| Rockow et al, 1983 | USA | 1 | Saline 4/11 5%IsoAlb 5/7 L-HES 2/7 | Hemodynamic parameters | In-hospital mortality |
| Rockow et al, 1989 | USA | 1 | 5%Iso-Alb5/10 H-HES 5/10 | Hemodynamic parameters | In-hospital mortality |
| Asfar et al, 2000 | France | 1 | H-HES 10/16 Gelatin 12/18 | Hemodynamic parameters | In-hospital mortality |
| Schortgen et al,2001 | France | 3 | H-HES 28/65 Gelatin 29/64 | Acute renal failure | In-hospital mortality |
| Finfer et al, 2004 | Aus. & NZ | 16 | Saline 217/615 4%IsoAlb 185/603 | Mortality | 28-day mortality |
| Molnar et al, 2004 | UK | 1 | H-HES12/15 Gelatin10/15 | Hemodynamic parameters | In-hospital mortality |
| Veneman et al, 2004 | Netherlands | 1 | Saline 5/16 20%Hyper-Alb 8/15 L-HES 18/30 | Plasma colloid pressure. | 30-day mortality |
| Palumbo et al, 2006 | Italy | 1 | 20%Hyper-Alb 3/10 L-HES 4/10 | Hemodynamic parameters. | 5-day mortality |
| Brunkhorst et al, 2008 | Germany | 18 | BC 93/274 H-HES 107/261 | Mortality | 90-day mortality |
| Friedman et al, 2008 | Belgium | 1 | 4%Iso-Alb5/15 H-HES 10/27 | Hemodynamic parameters. | In-hospital mortality |
| Li et al, 2008 | China | 1 | Saline 5/15 L-HES 9/15 | Mortality | 28-day mortality |
| McIntyre et al, 2008 | Canada and NZ | 4 | Saline 7/19 H-HES 9/21 | Feasibility trial. | 28-day mortality |
| Dolecek et al, 2009 | Czech Republic | 1 | 20%Hyper-Alb 4/30 L-HES 6/26 | Hemodynamic parameters. | In-hospital mortality |
| Dubin et al, 2010 | Argentina | 2 | Saline 7/13 L-HES 3/12 | Sublingual microcirculation. | 31-day mortality |
| Charpentier et al, 2011 | France | 29 | Saline 138/393 Hyper-Alb 138/399 | Mortality. | 28-day mortality. |
| Siegemund, 2011 | Netherlands | 1 | Hyper-Alb 50/124 L-HES 44/117 | Hemodynamic parameters. | In-hospital mortality |
| Guidet et al, 2012 | France and Germany | 24 | 6%HES 40/99 0.9%NaCI 32/95 | Volume for hemodynamic stable. | 90-days mortality |
| Myburgh et al, 2012 | Australia and NZ | 32 | 6%HES248/976  0.9%NaCl224/945 | Mortality | 90-days mortality |
| Perner et al, 2012 | Scandanavia | 26 | BC 173/400 L-HES 202/398 | Mortality | 90-days mortality |
| Annane et al, 2013 | Worldwide | 57 | BC16/37 Saline197/557 20%Hyper-Alb22/59 L-HES 120/375 Gelatin47/152 | Mortality | 90-days mortality |
| Caironi et al, 2014 | Italy | 100 | Saline 389/893 20%Hyper-Alb 365/888 | Mortality | 90-days mortality |
| Young et al,2015 | Aus. & NZ | 4 | BC 7/35 Saline 9/42 | Acute renal failure. | In-hospital mortality |
| Matthew et al, 2018 | USA | 1 | BC 418/2735 Saline 467/2646 | Mortality and acute kidney injury | 30-days mortality |
| Alasdair J. Gray,2024 | UK | 15 | 5% Human Albumin/Balanced Crystalloid 147/149 | recruitment rate | 30-day mortality. |
| Rithvik Golla ，2020 | USA | 1 | 0.9% Saline group  (n=80)/balance (n=80) | incidence of hyper  chloremia | In-hospital mortality |
| RakhiMaiwall 2022 | India | 1 | 20%Human Albumin/PlasmaLyte-148 50/50 | reversal of hypotension without initiation of vasopressors | 28-day mortality. |
| Surat Tongyoo 2020 | Thailand | 1 | Albumin/Gelatin 104/102 | all-cause mortality at 28 days | In-hospital mortality |
| Karen E.2020 | USA | 1 | BC / Saline 142/225 | 30-day in-hospital mortality | in-hospital mortality |
| Clarice 2019 | Brazil | 1 | 5% Human Albumin/Lactated Ringer 180/180 | death from any cause within  7 days | 28-day mortality. |
| Cyriac Abby 2020 | India | 1 | 5% Human Albumin/Saline 200/248 | MAP,≥65 mmHg] at 3 h. | 28-day mortality. |
| Fernando G.2021 | Brazil | 1 | BC / Saline | 90-day mortality. | 90-day mortality. |
| Simon Finfer 2022 | USA | 53 | BC / Saline | death from any cause  within 90 days | 1. day mortality. |
